# β-arrestin-dependent and -independent endosomal G protein activation by the vasopressin type 2 receptor

**DOI:** 10.1101/2023.04.01.535208

**Published:** 2023-04-02

**Authors:** Carole Daly, Akim Abdul Guseinov, Hyunggu Hahn, Irina G. Tikhonova, Alex Rojas Bie Thomsen, Bianca Plouffe

**Affiliations:** 1 Wellcome-Wolfson Institute for Experimental Medicine, School of Medicine, Dentistry and Biomedical Sciences, Queen’s University Belfast, Belfast, UK; 2 School of Pharmacy, Queen’s University Belfast, Belfast, UK; 3 Department of Molecular Pathobiology, New York University College of Dentistry, New York, USA; 4 NYU Pain Research Center, New York University College of Dentistry, New York, USA

## Abstract

The vasopressin type 2 receptor (V_2_R) is an essential GPCR in renal regulation of water homeostasis. Upon stimulation, the V_2_R activates Gα_s_ and Gα_q_/11, which is followed by robust recruitment of β-arrestins and receptor internalization into endosomes. Unlike canonical GPCR signaling, the β-arrestin association with the V_2_R does not terminate Gα_s_ activation, and thus, Gα_s_-mediated signaling is sustained while the receptor is internalized. Here, we demonstrate that this V_2_R ability to co-interact with G protein/β-arrestin and promote endosomal G protein signaling is not restricted to Gα_s_, but also involves Gα_q_/11. Furthermore, our data implies that β-arrestins potentiate Gα_s_/Gα_q_/11 activation at endosomes rather than terminating their signaling. Surprisingly, we found that the V_2_R internalizes and promote endosomal G protein activation independent of β-arrestins to a minor degree. These new observations challenge the current model of endosomal GPCR signaling and suggest that this event can occur in both β-arrestin-dependent and -independent manners.

## INTRODUCTION

The vasopressin type 2 receptor (V_2_R) is mainly known for its antidiuretic action in the kidney. Here, in the principal cells of the collecting duct, the V_2_R regulates water reabsorption from pre-urine by promoting translocation of water channel aquaporin 2 (AQP2) located in intracellular vesicles to the apical membrane^1^. The net result of this translocation is an enhanced water permeability. Defective V_2_R signaling due to loss or gain of function mutations is associated to nephrogenic diabetes insipidus^2^ or nephrogenic syndrome of inappropriate antidiuresis^3^, respectively.

The V_2_R belongs to the superfamily of G protein-coupled receptors (GPCRs), membrane proteins that control almost all physiological processes. Canonically, stimulation of GPCRs leads to coupling and activation of heterotrimeric G proteins (Gαβγ), which initiate downstream signaling cascades. GPCRs can couple to four families of Gα protein isoforms: Gα_s/olf_, Gα_i/o_, Gα_q/11_, and Gα_12/13_. Activation of each family leads to distinct downstream signaling events and cell biological outcomes. G protein activation is short lived and followed by receptor phosphorylation by GPCR kinases, which drives the recruitment of β-arrestins (βarrs) to the phosphorylated receptor. As βarrs interact with the same region of the receptor as G proteins, their recruitment physically uncouples G proteins from the receptor which causes desensitization of G protein signaling^4^. In addition, βarrs scaffold several proteins involved in endocytosis, which promotes receptor internalization into endosomes^5,6^.

Surprisingly, recent findings facilitated by the emergence of new molecular tools to interrogate signaling events with a subcellular resolution have challenged this plasma membrane centric view of G protein signaling. Several GPCRs, including the V_2_R, have been reported to engage in G protein signaling after receptor internalization into early endosomes and/or other intracellular compartments^7,8^. Interestingly, endosomal Gα_s_ signaling by V_2_R was demonstrated to enhance sustained translocation of AQP2 to the plasma membrane to facilitate water reabsorption^7^. This endosomal stimulation of G protein signaling by barr-bound GPCRs has been difficult to reconcile with the aforementioned canonical understanding of GPCR signaling since G protein and barr interactions with GPCRs were thought to be mutually exclusive. However, we discovered and delineated a new signaling paradigm whereby some GPCRs, including the V_2_R, bind βarrs in a specific manner; in this conformation, βarr only interacts with the receptor carboxy-terminal tail thereby permitting the receptor transmembrane core to bind with G proteins simultaneously to form a “megaplex”^8–10^. Due to the simultaneous engagement with G protein and βarr, the receptor in these megaplexes maintains its ability to activate G protein, even while being internalized by βarrs.

Although known as a Gα_s_-coupled receptor, several studies report activation of the Gα_q/11_ isoforms by V_2_R^11–15^ as well as unproductive coupling to Gα_12_^14^. Therefore, we hypothesized that the V_2_R form megaplexes with both Gα_s_ and Gα_q_ leading to endosomal activation of both Gα_s_ and Gα_q_. In addition, pulse-stimulation experiments of the V_2_R and parathyroid hormone type 1 receptor (PTHR) demonstrated that sustained Gα_s_-mediated signaling was enhanced by βarr^7,16^. To address whether such βarr-mediated increase in G protein signaling is a result of direct coupling and activation of G proteins at endosomes, we here applied a combination of approaches based on engineered mini G proteins (mG proteins)^17,18^, enhanced bystander bioluminescence resonance energy transfer (EbBRET)^19^, nanoluciferase binary technology (NanoBiT)^20^, and confocal microscopy imaging.

## RESULTS

### The V_2_R activates Gα_s_ and Gα_q_ from early endosomes

**Fig. 1:**
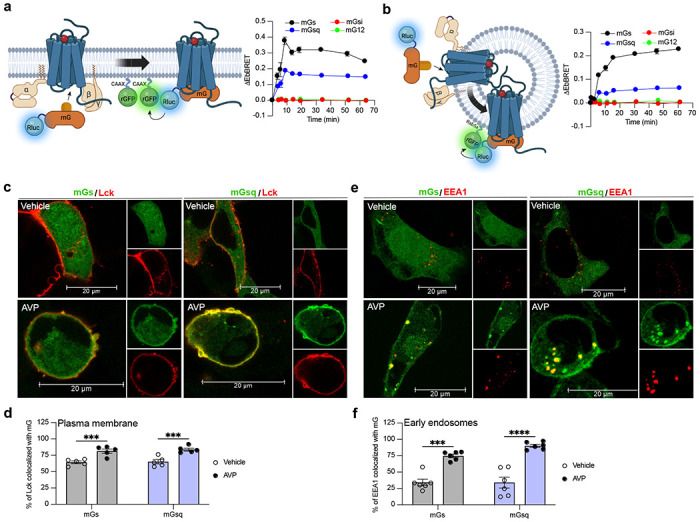
Activation of Gα_s_ and Gα_q_ at plasma membrane and early endosomes upon AVP stimulation. **a,** Left: Illustration of EbBRET-based biosensors used to monitor G protein activation by the V_2_R at the plasma membrane. Right: Kinetics of the recruitment of mG proteins at the plasma membrane upon stimulation of V_2_R-expressing HEK293 cells with 1 μM AVP. See also Supplementary Fig. 1a for expression of each mG construct. **b,** Left: Illustration of EbBRET-based biosensors used to monitor G protein activation by the V_2_R from early endosomes. Right: Kinetics of the recruitment of mG proteins to early endosomes upon stimulation of V_2_R-expressing HEK293 cells with 1 μM AVP. See also Supplementary Fig. 1b for the expression of each mG construct. *n* = 3 biological replicates for mGs, mGsi, and mGsq, and *n* = 4 for mG12 for a and b. **c,** Confocal microscopy of HEK293 cells expressing RFP-Lck, V_2_R, and Halo-mGs (left panels), or Halo-mGsq (right panels) stimulated for 10 minutes with vehicle (upper panels) or 1 μM AVP (bottom panels). **d,** Scatter plots showing Lck/mG colocalization performed on 5 representative images. **e,** Confocal microscopy of HEK293 cells expressing RFP-EEA1, V_2_R, and Halo-mGs (left panels), or Halo-mGsq (right panels) stimulated for 45 minutes with vehicle (upper panels) or 1 μM AVP (bottom panels). **f,** Scatter plots showing EEA1/mG colocalization performed on 6 representative images. Asterisks mark statistically significant differences between vehicle and AVP treatments as assessed by two-way ANOVA and Sidak’s post hoc test for multiple comparisons (***P ≤ 0.001, ****P ≤ 0.0001). All data are presented as mean ± s.e.m.

To measure the activation of the four families of G protein isoforms at the plasma membrane and early endosomes by the V_2_R in real-time, we used mG proteins. The mG proteins are homogenously distributed in the cytosol under basal condition but translocate to the subcellular location of GPCRs upon stimulation^17,18,21^. In addition, we applied an EbBRET approach instead of a conventional bioluminescence resonance energy transfer (BRET)-based assay to monitor mG protein trafficking. EbBRET displays superior robustness and sensitivity, as well as higher dynamic spectrometric energy transfer signals associated to EbBRET as compared to conventional BRET, which is why this approach was favored^19^. We fused mG proteins to the luciferase from *Renilla reniformis* (Rluc) and anchored green fluorescent protein from the same species (rGFP) to polybasic sequence and prenylation CAAX box of KRas (rGFP-CAAX), which is located at the plasma membrane^22^, or to the early endosome marker Rab5^23^ (Fig. 1a,b, left panels). Four variants of mG proteins (mGs, mGsi, mGsq, and mG12) have been designed and shown to maintain the receptor-Gα protein specificity of the four Gα subunit isoform families^18^. In HEK293 cells expressing rGFP-CAAX, V_2_R, and similar levels of Rluc-fused mG proteins (Supplementary Fig. 1a), arginine vasopressin (AVP) treatment induced a rapid recruitment of mGs and mGsq but not mGsi nor mG12 to the plasma membrane. Maximal recruitment of the mGs and mGsq were reached ~10 minutes after initial stimulation (Fig. 1a, right panel). These results suggest that the V_2_R activates both Gα_s_ and Gα_q_ at the plasma membrane. In addition, AVP stimulation led to the recruitment of the same mG protein isoforms to early endosomes in cells expressing rGFP-Rab5, V_2_R, and similar levels of Rluc-fused mG proteins (Fig. 1b, right panel and Supplementary Fig. 1b). In contrast to the plasma membrane response, mGs and mGsq recruitment to early endosomes were slower and reached maximal levels 45-60 minutes after initial stimulation with AVP (Fig. 1b, right panel).

To visualize V_2_R-mediated activation of Gα_s_ and Gα_q_ at the plasma membrane and early endosomes we used confocal microscopy. For this purpose, we transfected HEK293 cells with mGs or mGsq fused to a HaloTag (Halo-mGs and Halo-mGsq) along with the respective red fluorescent protein (RFP)-fused plasma membrane or early endosomes markers Lck^24^ or early endosome antigen 1 (EEA1)^25^. Upon HaloTag labelling with a fluorescent green ligand, mGs and mGsq were visible and homogenously distributed in the cytosol under basal condition (vehicle) (Fig. 1c). In contrast, in cells treated with AVP for 10 minutes, both mGs and mGsq were redistributed along the periphery of the cells where they colocalized with RFP-Lck (Fig. 1c,d). These observations confirm that Gα_s_ and Gα_q_ are activated by the V_2_R at the plasma membrane. In cells expressing the early endosomal marker RFP-EEA1, robust colocalization between the Halo-mG proteins and RFP-EEA1 were found 45 min after initial AVP-stimulation but not by vehicle treatment (Fig. 1e,f). Together our EbBRET and confocal microscopy imaging data suggest that Gα_s_ and Gα_q_ are activated by V_2_R first at plasma membrane, and later on, from early endosomes after the V_2_R has been internalized.

### The V_2_R recruits Gα_s_/Gα_q_ and βarrs simultaneously

G protein activation from endosomes by some GPCRs is associated with the ability of the receptor to recruit G protein βarr and simultaneously to form a GPCR-βarr-G protein megaplex. As we already previously demonstrated that the V_2_R forms V_2_R-βarr-G_s_ megaplexes upon AVP stimulation^8^, we here explored whether formation of such complexes potentially can be formed with G_q/11_ as well. In addition to the V_2_R, we also applied a chimeric V_2_R harboring the carboxy-terminal tail of the β2-adrenergic receptor (β_2_AR) referred to as V_2_β_2_AR. We previously showed that the phosphorylated V_2_R carboxy-tail forms stable complexes with βarr, a requirement of megaplex formation, whereas the carboxy-tail of the β_2_AR does not^10^. Therefore, we expected that only the V_2_R, but not the V_2_β_2_AR, recruits G proteins and βarrs simultaneously upon agonist challenge.

**Fig. 2:**
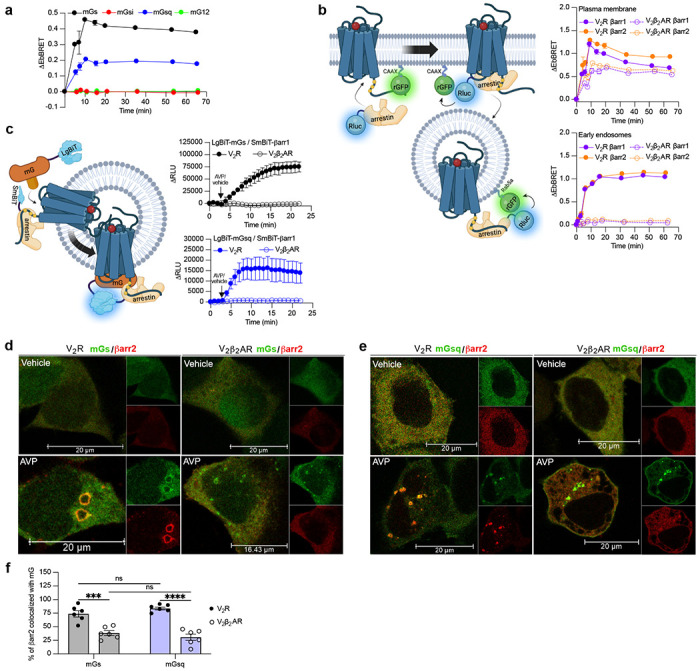
Formation of megaplexes with Gα_s_ or Gα_q_ upon stimulation of V_2_R with AVP. **a,** Kinetics of mG protein recruitment to the plasma membrane upon stimulation of V_2_β_2_AR with 1 μM AVP. See Supplementary Fig. 2 for expression of each mG construct. *n*=3 for mGs, mGsi, and mGsq, and *n*=4 for mG12. **b,** Left: Illustration of EbBRET biosensors used to monitor βarr recruitment to the plasma membrane and endosomes. Right: Kinetics of the recruitment of βarr1 and βarr2 to the plasma membrane (upper panel) and to early endosomes (bottom panel) upon stimulation of V_2_R or V_2_β_2_AR with 1 μM AVP. See Supplementary Fig. 3 for the relative expression of V_2_R or V_β_P_2_AR at plasma membrane and of βarrs. *n*=3 for all conditions. **c,** Left panel: Illustration of nanoBiT biosensors used to monitor simultaneous coupling of Gα proteins and βarr1 to GPCRs. Right panels: Kinetics of the proximity between SmBiT-βarr1 and LgBiT-mGs (upper panel) or LgBiT-mGsq (bottom panel) upon stimulation of the V_2_R or V_2_β_2_AR with 1 μM AVP. *n*=3 for all conditions. **d,e,** Confocal microscopy of HEK293 cells expressing Halo-mGs or Halo-mGsq, strawberry-βarr2, and V_2_R (left panels), or V_2_β_2_AR (right panels). The cells were stimulated for 45 minutes with vehicle (upper panels) or 1 μM AVP (bottom panels). **f,** Scatter plots of the percentage of βarr2 colocalization with mGs or mGsq upon stimulation with 1μM AVP (6 representative images). Asterisks mark significant differences between V_2_R and V_2_β_2_AR assessed by two-way ANOVA and Sidak’s post hoc test for multiple comparisons (***≤0.001, ****≤0.0001). No statistical difference (ns) was detected between mGs and mGsq for V_2_R (*P*=0.6640) and V_2_β_2_AR (*P*=8446). Data are presented as mean ± s.e.m.

Both the V_2_R and V_2_β_2_AR bind to AVP with similar affinities and activate adenylyl cyclase via Gα_s_ with similar potencies^26^. We monitored activation of the four Gα protein families at the plasma membrane by the V_2_β_2_AR upon AVP treatment using the same approach utilized in Fig. 1a. Similarly to the V_2_R, the V_2_β_2_AR activated both Gα_s_ and Gα_q_, but not Gα_i_ or Gα_12_, at plasma membrane with a maximal response reached after ~10 minutes of stimulation (Fig. 2a, and Supplementary Fig. 2). While the V_2_R and V_2_β_2_AR are both reported to internalize via a βarr-dependent mechanism, βarr has been reported to rapidly dissociate from the V_2_β_2_AR shortly after its recruitment to the plasma membrane due to its low affinity for this receptor chimera^26^. In contrast, βarr stays associated with the V_2_R during its internalization into endosomes owning to its high affinity for the V_2_R^26^. Here, we compared the kinetics of βarr1 and βarr2 recruitment to the V_2_R and V_2_β_2_AR at the plasma membrane and early endosomes by monitoring AVP-promoted EbBRET between Rluc-fused βarrs and rGFP-CAAX or rGFP-Rab5, respectively (Fig. 2b, left panel). At similar levels of receptor and βarr expressions (Supplementary Fig. 3a), both receptors recruited βarr1 and βarr2 at plasma membrane maximally after 10 minutes of stimulation with AVP (Fig. 2b, right upper panel). However, the presence of βarrs at the plasma membrane declined rapidly hereafter 10 minutes in V_2_R-expressing cells, while remaining for longer periods of time in V_2_β_2_AR-expressing cells. These findings are in line with the previous reported observations^26^. Additionally, the translocation of βarrs to the plasma membrane was more robust for the V_2_R as compared to the V_2_β_2_AR, which is reminiscent from the higher affinity of βarrs for the V_2_R as compared to the β_2_AR^27^. In contrast to the rapid translocation of βarrs to the plasma membrane, AVP treatment induced a robust but slower recruitment of βarrs to early endosomes with V_2_R reaching a maximal response after approximately 45 minutes of stimulation (Fig. 2b, right bottom panel). Importantly, as opposed to V_2_R, AVP-stimulation of V_2_β_2_AR did not result in βarr translocation to early endosomes (Fig. 2b, right bottom panel, and Supplementary Fig. 3b). Consequently, the V_2_β_2_AR represents a valuable negative control to investigate the ability to recruit G proteins and βarrs simultaneously at endosomes.

To track the simultaneous coupling of G proteins and βarrs to GPCRs in real-time, a nanoBiT approach was used. Both mGs and mGsq were fused to the large portion of nanoluciferase (large-BiT; LgBiT) and βarr1 to an optimized small peptide BiT (small BiT; SmBiT). Reconstitution of the complete and functional nanoluciferase, which catalyzes the conversion of coelenterazine h, results in emission of a bright luminescence signal. In our setup, close proximity of LgBiT and SmBiT only occurs when LgBiT-mG and SmBiT-βarr1 are recruited simultaneously to the receptor, which is a hall mark of megaplex formation (Fig. 2c, left panel). Using this approach, we detected bright luminescence signals involving mGs/βarr1 (Fig. 2c, upper right panel) and mGsq/βarr1 (Fig. 2c, bottom right panel) upon stimulation of the V_2_R but not the V_2_β_2_AR. Interestingly, the dual coupling of Gα_q_/βarr to V_2_R appeared to be faster than the co-coupling of Gα_s_/βarr. While 20 minutes was required to reach the maximal response of V_2_R-stimulated mGs/βarr1 co-coupling, 8 minutes was sufficient to obtain the maximal levels of mGsq/βarr1 recruitment to the V_2_R (Fig. 2c, right panels).

To visualize the simultaneous recruitment of G proteins and βarr by confocal microscopy, we transfected HEK293 cells with βarr2 fused to RFP (RFP-βarr2), Halo-mGs or Halo-mGsq, and the V_2_R or V_2_β_2_AR. In vehicle-treated cells, both mGs and βarr2 were homogenously distributed in the cytosol (Fig. 2d). However, after prolonged stimulation of V_2_R with AVP, around 75% of βarr2 colocalized with mGs in endocytic vesicles (Fig. 2d,f). In V_2_β_2_AR-stimulated cells, little to no colocalization was observed between βarr2 and mGs upon prolonged stimulation with AVP (Fig. 2d, f). Surprisingly, however, some clusters of intracellular mGs were clearly visible (Fig. 2d, f). These results suggest simultaneous coupling of Gα_s_/βarr2 to the V_2_R in endosomes but not to the V_2_β_2_AR. In cells expressing mGsq, both mGsq and βarr2 were also homogenously distributed in the cytosol when cells were treated with the vehicle in a similar fashion to cells expressing mGs (Fig. 2e). Upon AVP stimulation, approximately 75% of βarr2 colocalized with mGsq in intracellular vesicles in V_2_R-expressing cells, whereas poor colocalization was observed in V_2_β_2_AR-expressing cells (Fig. 2e,f). However, similarly to cells expressing mGs, some clusters of intracellular mGsq were visible in cells expressing V_2_β_2_AR, suggesting a certain level of endosomal Gα_s_/Gα_q_ signaling despite the absence of βarr2.

### βarr-dependent and -independent endosomal G protein activation by the V_2_R

**Fig. 3:**
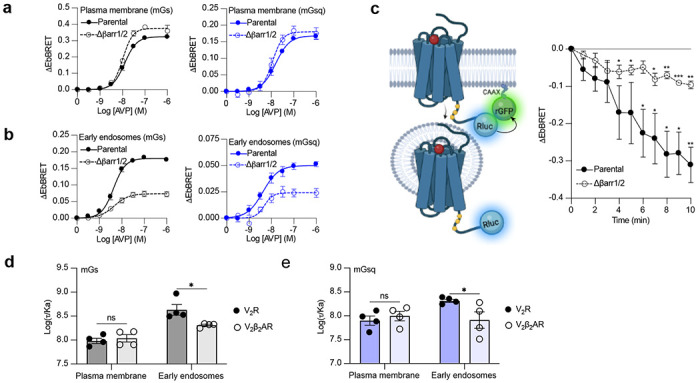
Contribution of megaplex to endosomal Gα_s_ and Gα_q_ signaling. **a,b,** AVP dose-response curves of the recruitment of mGs (left panel) and mGsq (right panel) to the plasma membrane and early endosomes using parental or Δβarr1/2 cells. The cells were stimulated for 10 minutes (plasma membrane) or 45 minutes (early endosomes) with AVP. See Supplementary Fig. 4a,b for relative V_2_R expression levels at the plasma membrane in parental and Δβarr1/2 cells. *n*=4 biological replicates for each condition. See also Supplementary Table 1 for parameters related to dose response curves. **c,** Left: Illustration of EbBRET biosensors used to monitor AVP-mediated internalization of the V_2_R. Right: Kinetics of V_2_R internalization upon stimulation with AVP 0.1 μM. See Supplementary Fig. 4c for relative expression of V_2_R in parental and Δβarr1/2 cells. n=4 and asterisks mark significant differences from zero as assessed by one sample t test (*≤0.05, **≤0.01, ***≤0.001). **d,e,** Transduction coefficients of mGs and mGsq recruitment to the plasma membrane and early endosomes in V_2_R- or V_2_β_2_AR-expressing HEK293 cells. The cells were stimulated 10 minutes (plasma membrane) or 45 minutes (early endosomes) with 1 μM AVP. See Supplementary Fig. 5 and Supplementary Table 2 for the dose-response curves and associated parameters, respectively. See also Supplementary Fig. 4d,e for the relative expressions of V_2_R and V_2_β_2_AR at the plasma membrane. Asterisks mark significant differences between the V_2_R and V_2_β_2_AR as assessed by two-way ANOVA and Sidak’s post hoc test for multiple comparisons (*≤0.05). No statistical difference (ns) was detected between the V_2_R and V_2_β_2_AR for mGs (*P*=0.7692) and mGsq (*P*=0.7445) at the plasma membrane. *n*=4 for each condition. All data are presented as mean ± s.e.m.

Activation of Gα_s_ and Gα_q_ by the V_2_β_2_AR from endosome-like structures in the absence of local βarr raises the possibility that the V_2_R can activate these G proteins from endosomes in both βarr-dependent and -independent manners. To test this hypothesis, we compared AVP-induced Gα_s_ and Gα_q_ activation at plasma membrane and endosomes in CRISPR/Cas9-engineered βarr1- and βarr2-deficient HEK293 cells (Δβarr1/2)^19^ as well as their parental cellular counterpart. The surface expression of V_2_R was matched in both cellular backgrounds (Supplementary Fig. 4a,b). Using the EbBRET biosensors described in Fig. 1a-b, we performed AVP concentration-response characterization of Gα_s_ and Gα_q_ activation at the plasma membrane (Fig. 3a) and early endosomes (Fig. 3b) in parental and Δβarr1/2 HEK293 cells. In contrast to Gα_s_ and Gα_q_ activation at the plasma membrane, which were not negatively affected by the absence of βarrs (Fig. 3a), we observed a robust decrease in the ability of the V_2_R to activate Gα_s_ and Gα_q_ at endosomes in Δβarr1/2 cells as compared to their parental counterpart (Fig. 3b and Supplementary Table 1). These data demonstrate the important role of βarrs in endosomal Gα_s_/Gα_q/11_ activation by the V_2_R. However, although the Δβarr1/2 cells do not express βarrs, we still observed significant residual G protein activation from endosomes. This surprising observation suggests that the V_2_R internalizes into endosomes to some extent in a βarr-independent manner from where G proteins are stimulated. To probe this possibility, we compared V_2_R internalization in parental and Δβarr1/2 HEK293 cells expressing rGFP-CAAX and equivalent amounts of V_2_R fused to Rluc at its carboxy-terminal tail (V_2_R-Rluc) (Fig. 3c, left panel and Supplementary Fig. 4c). In parental HEK293 cells, AVP-stimulation of the V_2_R-Rluc led to a robust decrease of EbBRET values, which indicates strong receptor internalization (Fig. 3c, right panel). Interestingly, we also observed significant internalization of the V_2_R in Δβarr1/2 HEK293 cells, although less than in the parental cells (Fig. 3c, right panel). These results suggest that a minor population of V_2_R internalizes independently of βarrs and contributes to endosomal Gα_s_ and Gα_q/11_ signaling.

### βarrs potentiate endosomal Gα_s_ and Gα_q_ activation by the V_2_R.

Although our data suggest that a minor population of V_2_R internalizes in the absence of βarrs and contribute to V_2_R-mediated endosomal Gα_s_ signaling, it has been reported that βarr binding to the V_2_R and parathyroid hormone receptor (PTHR) potentiates endosomal Gα_s_ signaling^7,16^. To verify this and determine if this potentiator effect of βarrs also affects endosomal Gα_q/11_ signaling, we compared endosomal Gα_s_ and Gα_q/11_ activation in cells expressing similar levels of V_2_R or V_2_β_2_AR (Supplementary Fig. 4d,e). Our rationale for using these two receptors is that if βarrs potentiate endosomal G protein activation, this potentiator effect will be observed to a greater extent for the V_2_R since this receptor associates more robustly with βarrs as compared to the V_2_β_2_AR. Using the same biosensors as in Fig. 1a-b, we performed AVP dose-response curves of mGs and mGsq recruitment to the plasma membrane and early endosomes (Supplementary Fig. 5, and Supplementary Table 2). From the dose-response curves obtained (Supplementary Fig. 5, and Supplementary Table 2), we determined the transduction coefficient log(τ/Ka), a parameter that combines efficiency and potency to determine the overall G protein transduction, for each condition using the operational model of Kenakin and Christopoulos^28^. In cells expressing mGs, the transduction coefficients of Gα_s_ activation at the plasma membrane were similar for the V_2_R and V_2_β_2_AR, but higher for the V_2_R than V_2_β_2_AR in early endosomes (Fig. 3d and Supplementary Table 2). Similarly, in cells expressing mGsq, the transduction coefficients of Gα_q/11_ activation at the plasma membrane were similar for the V_2_R and V_2_β_2_AR, but higher for the V_2_R than V_2_β_2_AR in early endosomes (Fig. 3e and Supplementary Table 2). Altogether these results indicate that βarrs potentiate activation of G proteins by the V_2_R in early endosomes.

## DISCUSSION

In the present work, we addressed the spatial aspect of G protein signaling by the V_2_R and investigated the potential role of βarrs in modulating these responses. Several studies report activation of Gα_s_ and Gα_q/11_ by the V_2_R using a wide range of assays^11–15^. However, these assays lack spatial resolution or are measured by default at the plasma membrane. Here, we demonstrated that both Gα_s_ and Gα_q/11_ are activated by the V_2_R at the plasma membrane as well as early endosomes using a mG proteins-based approach. The PTHR, a GPCR that regulates mineral ion homeostasis and bone development, also couples to both Gα_s_ and Gα_q/11_^29^. Similar to our observations of the V_2_R, the reduction of PTHR internalization by βarr1 and βarr2 depletion strongly decreases endosomal Gα_s_/cAMP signaling. However, in contrast to the V_2_R, βarr-mediated receptor internalization shuts down Gα_q/11_-mediated responses, and thus, the PTHR does not appear to stimulate Gα_q/11_ from endosomes^16,30^.

The reason for this inability of internalized PTHR to activate Gα_q/11_ from endosomes is not known. However, it is unlikely to be a general feature of these G protein isoforms as multiple laboratories have reported endosomal GPCR signaling events downstream of Gα_q/11_ activation. These events include measurements of signal-amplified responses such as protein kinase C (PKC) recruitment or ERK1/2 activation^31–33^. Recently, direct activation of Gα_q/11_ from early endosomes was monitored using a mG protein-based approach and effector membrane translocation assay (EMTA)^34^. In this study, Wright *et al.* demonstrated that stimulation of Gα_q/11_ protein isoforms by receptors at the plasma membrane does not necessarily lead to the activation of the exact same isoforms at endosomes. For example, the authors showed that the thromboxane A_2_ alpha isoform receptor (TPαR) robustly activates all the Gα_q/11_ isoforms (Gα_q_, Gα_11_, Gα_14_, and Gα_15_) at the plasma membrane, but only activates Gα_q_ and Gα_11_ isoforms at endosomes. In contrast, the muscarinic acetylcholine M_3_ receptor (M_3_R) activates all four Gα_q/11_ isoforms both at plasma membrane and endosomes. While G protein selectivity at plasma membrane is mainly dependent on receptor conformation^35,36^, specific residues present at the GPCR-Gα protein interface^37^, as well as the location and duration of these intermolecular interactions^38^, endosomal G protein activation seems to be controlled by additional factors that are not fully understood.

The presence of serine/threonine phosphorylation site clusters at the carboxy-terminal tail of GPCRs delineates two major classes of receptors; class A and class B^27^. Class A GPCRs such as the β_2_AR are defined by harboring few single phosphorylation sites, which form interactions with positively charged residues of βarrs. In addition to the phosphorylated receptor residues, the class A GPCR–βarr association also depends on an interaction between the βarr fingerloop region and the receptor transmembrane core, which sterically block G protein access to the GPCR^10,39^. The class A GPCR-βarr association is transient and the complex dissociates shortly after endocytosis, which results in receptor recycling back to the cell surface. In contrast, class B GPCRs including the V_2_R are defined by having phosphorylation site clusters in the carboxy-terminal tail that form highly stable associations with βarrs solely through this region. This strong interaction leads to prolonged receptor internalization into endosomes^10^. As the stability of this GPCR-βarr complex ‘tail’ conformation does not depend on the interaction between the βarr fingerloop region and the receptor core, the GPCR can internalize via βarrs into different intracellular compartments while stimulating G protein signaling for prolonged periods of time^7,8,10,40,41^. Previously, formation of such GPCR-G protein-βarr megaplexes at intracellular compartments has only been reported with Gα_s_ or Gα_i/o_ proteins^8,42,43^. In the present study we demonstrate that megaplex formation is not confined to these G protein isoforms but also appears to form with other G protein isoforms such as Gα_q/11_.

An interesting aspect of βarr/megaplex-dependent endosomal G protein signaling is whether βarrs only acts a vehicle that transports GPCRs to this subcellular location from where they activate G proteins or whether βarrs in megaplexes themselves directly modulate G protein activity. In the current study, we show that βarrs directly potentiate G protein activation by the V_2_R in early endosomes (Fig. 3). These findings are further supported by Feinstein *et al.* who previously demonstrated that V_2_R-stimulated G protein activation is positively modulated by the presence of βarr2^7^. However, in the recent cryo-electron microscopy high-resolution structure of an engineered class B GPCR-Gs-βarr1 megaplex, no direct interaction between the heterotrimeric Gs and βarr1 was observed, and thus, it is not obvious how βarrs may affect G protein activity from this structure^9^. On the other hand, biochemical studies of the megaplex and G protein-βarrs interactions demonstrated that βarr can serve as a scaffold for the Gβγ subunits that are released upon activation of the heterotrimeric G protein^8,44,45^. Thus, this Gβγ scaffolding role of βarr may confine Gα_s_ and Gα_q/11_ near endosomally-located V_2_R, leading to their re-activation as soon as the inactive GDP-bound Gα with Gβγ subunits reassemble. The results of such activation mechanism would be a net increase in the G protein activation rate.

Surprisingly, our results using Δβarr1/2 cells indicate that the V_2_R not only promote endosomal G protein signaling in a βarr/megaplex-dependent manner but also can internalize and activate G proteins from endosomes in a βarr-independent fashion (Fig. 3a,b). Although our data showed that βarr-independent endosomal G protein activation is substantial less effective than the βarr-dependent mechanism for the V_2_R, it still represents an alternative mode of endosomal GPCR signaling that little is known about. Interestingly, in a very recent study of the vasoactive intestinal peptide receptor 1 (VIPR1) by Blythe & von Zastrow, it was shown that VIPR1 promotes robust G protein signaling from endosomes and that this occurs in a completely βarr-independent fashion^46^. Perplexingly, the authors observed that agonist-stimulation of VIPR1 led to recruitment of βarr1 and receptor internalization into endosomes where VIPR1 and βarr1 colocalized. However, despite this potential interaction between VIPR1 and βarrs, the presence of βarr1/2 had little to no effect on receptor internalization and the ability of VIPR1 to activate G protein from endosomes^46^. As two independent studies using two different receptor systems now have found that endosomal G protein signaling can be achieved independent of βarrs, it is likely that this alternative mode of signaling represents a more general mechanism that is utilized by multiple GPCRs to regulate important physiological functions. Thus, further investigation into the details of βarr-independent receptor internalization and endosomal G protein signaling is much needed.

**Fig. 4:**
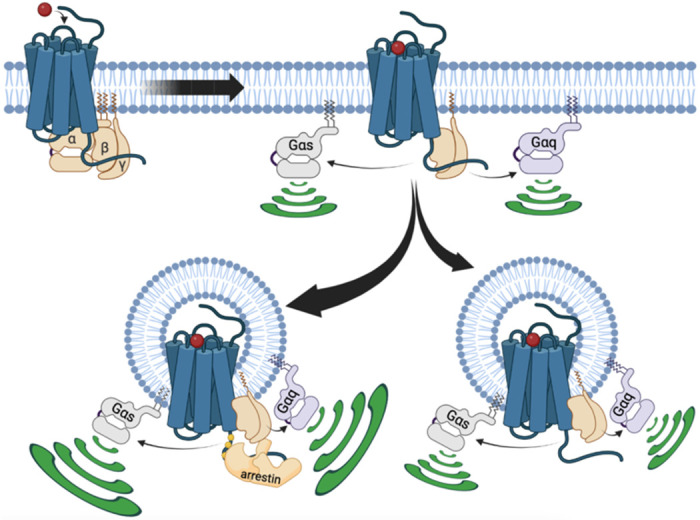
Updated model of V_2_R signaling. At the plasma membrane, AVP binding to V_2_R results in receptor-mediated Gα_s_ or Gα_q_ activation. This initial G protein activation at the plasma membrane is followed by V_2_R internalization into early endosomes. This internalization occurs primarily in a βarr-dependent manner, leading to the formation of a megaplex with Gα_s_ or Gα_q/11_ and robust activation of these G proteins from endosomes. Additionally, a minor population of V_2_R internalize in a βarr-independent fashion, which also leads to minor but significant Gα_s_ or Gα_q/11_ activation from endosomes.

In summary, in the present study we gain new insights into how internalized V_2_R stimulates G protein signaling from endosomes, which require us to modify the current model (Fig. 4). We demonstrated that V_2_R-mediated endosomal G protein activation is not restricted to the Gα_s_ isoform but also occurs with the Gα_q/11_ isoforms. A major part of this endosomal G protein activation is βarr-dependent, and presumably takes place through the formation of V_2_R–G protein–βarr megaplexes. Interestingly, the presence of βarrs in these megaplexes potentiates the ability of the V_2_R to activate G protein within endosomes. Surprisingly, we found that this mechanism is not the only way internalized V_2_R stimulates G protein signaling from endosomes since this event can take place in a completely βarr-independent fashion as well. The underlying details of how βarr-independent endosomal G protein activation by the V_2_R takes place is not known. However, since similar observation were made in another study of the VIPR1, the mechanism might represent a general aspect of GPCR biology that control important physiological and pathophysiological processes.

## METHODS

### Cell culture and transfection

HEK293 clonal cell line (HEK293SL cells) and referred as HEK293 cells as well as the HEK293 cells devoid of βarr1 and βarr2 referred as Δβarr1/2 cells were a gift from Stephane Laporte (McGill University, Montreal, Quebec, Canada) and previously described^19^. These cells were cultured in Dulbecco’s Modified Eagle’s Medium (DMEM) high glucose (Gibco) supplemented with 10% fetal bovine serum and 100 units per ml penicillin-streptomycin (Gibco), maintained at 37^°^C and 5% CO_2_ and passaged every 3-4 days using trypsin-EDTA 0.05% (Gibco) to detach the cells. DNA to be transfected was combined with salmon sperm DNA (Invitrogen) to obtain a total of 1 μg DNA per condition. Linear polyethyleneimine 25K (PEI; Polysciences) was combined with DNA (3 μg PEI per μg of DNA), vortexed and incubated 20 minutes before adding a cell suspension containing 300,000 cells per ml (1.2 ml of cells per condition). The appropriate volume of cells containing the DNA was seeded and cells were incubated for 48 hours before assay.

### DNA plasmids

All DNA constructs were cloned into pcDNA3.1(+) expression plasmid except if stated otherwise. V_2_R and V_2_β_2_AR were tagged with a HA epitope in amino-terminal of the receptors. HA-V_2_R was synthetized by GenScript and HA-V_2_β_2_AR was generously provided by Dr Robert Lefkowitz (Duke University, USA). C-tRFP-Lck (cloned into PCMV6-AC-RFP expression vector) and TagRFP-T-EEA1 (cloned into pEGFP-C1 vector) were purchased from Addgene (respectively #RC100049 and #42635). Strawberry-tagged βarr2 was a gift from Prof. Marc G. Caron (Duke University, USA). rGFP-CAAX^19^, rGFP-Rab5^19^, V_2_R-Rluc^19^, Rluc-βarr1^47^, Rluc-βarr2^48^ were previously described. Rluc-mGs, Rluc-mGsi, Rluc-mGsq, and Rluc-mG12 were synthetized by Twist Bioscience and cloned into pTwistCMV expression vector. The Venus tag in NES-Venus-mGs, NES-Venus-mGsi, NES-Venus-mGsq, and NES-Venus-mG12 previously described^18^ was replaced by Rluc. Halo-mGsq was kindly provided by Prof. Nevin A. Lambert (Augusta University, USA). LgBiT-mGsq and SmBiT-βarr1 were synthetized by GenScript. mGsq and βarr1 were tagged in amino-terminal with LgBiT and a linker peptide and SmBiT, respectively.

### Enhanced bystander Bioluminescence Resonance Energy Transfer (EbBRET) assays

The cell suspension containing DNA (EbBRET biosensors and receptors) were seeded in white 96-well plates (Greiner) at 30,000 cells/well (100 μl per well). 48 hours after transfection, cells were washed with DPBS (Gibco) and assayed in Tyrode’s buffer containing 137 mM NaCl, 0.9 mM KCl, 1 mM MgCl_2_, 11.9 mM NaHCO_3_, 3.6 mM NaH_2_PO_4_, 25 mM Hepes, 5.5 mM glucose, 1 mM CaCl_2_ (pH 7.4) at 37^°^C. AVP or vehicle (water) were added and cells incubated at 37^°^C for the required time. 5 or 15 minutes before reading, 2.5 μM of the Rluc substrate coelenterazine 400a or 1.33 μM of methoxy e-coelenterazine (NanoLight Technology) was added, respectively. All EbBRET measurements were performed using a FLUOstar Omega microplate reader (BMG Labtech) with an acceptor filter (515 ± 30 nm) and donor filter (410 ± 80 nm). EbBRET values were determined by calculating the ratio of the light intensity emitted by the acceptor over the light intensity emitted by the donor. In kinetics or dose-response curves, ΔEbBRET is defined as the values of EbBRET in presence of AVP minus the value obtained with vehicle. Dose-response curves were fitted using nonlinear regression using a 4-parameter equation and the basal ΔEbBRET was fixed to zero. Statistical significance of parameters of dose-response curves (AVP-induced maximal efficacy or potency) was established by comparing independent fits with a global fit that shares the selected parameter using extra sum-of-squares F test. The transduction coefficients Log(τ/Ka) were determined using the operational from Kenakin and Christopoulos as previously described^49^.

### NanoBiT assay

The NanoBiT assay to measure proximity between LgBiT-mG proteins and SmBiT-βarr1 has been reported previously^50^. In short, 2,000,000 cells were seeded per well in 6 well plates. 24 hours later, 125 ng SmBiT-βarr1, 1000 ng V_2_R or V_2_β_2_AR, and 125 ng LgBiT-mGs or 1000 ng LgBiT-mGsq were transfected into the cells using Lipofectamine 3000 transfection reagent. The next day, transfected cells were detached and 100,000 cells/well were plated into a Poly-D-lysine-coated white 96-well Microplate (Falcon) and incubated overnight at 37°C. The cells were equilibrated in Opti-MEM at 37°C for 60 minutes. Coelenterazine-h was added at a final concentration of 10 μM before starting the measurement. After establishing a baseline response for 2 minutes, cells were stimulated with AVP added at a final concentration of 100 nM and the luminescence was measured for additional 20 minutes. The signal was detected at 550 nm using a PHERAstar *FSX* instrument (BMG LabTech). ΔRLU is defined as the values of relative luminescence in presence of AVP minus the value obtained with vehicle.

### Confocal microscopy

Cells containing DNA (fluorescent-tagged localization markers, Halo-mGsq, and receptors) were seeded in 8-well glass chambered slides (Ibidi GMBH) at 30,000 cells per well. The day of the assay, HaloTag^®^ Oregon Green^®^ Ligand (Promega) was added to cells at a final concentration of 1 μM in the culture media and incubated 15 minutes (37°C, 5% CO_2_) to label Halo-mGsq. Cells were washed 3 times with the media and incubated 30 minutes (37°C, 5% CO_2_) for the last wash. The media was aspirated, replaced by Tyrode’s buffer and cells were stimulated with AVP or vehicle (water) for the required time at 37°C, 5% CO_2_. At the end of the incubation, the media was aspirated and cells were fixed by adding 300 μl per well of 4% paraformaldehyde in PBS (Thermo Scientific) and incubated at room temperature for 10 minutes. The paraformaldehyde solution was aspirated, replaced by DPBS and cells were incubated for 10 minutes before being replaced by Tyrode’s buffer (300 μl per well) and visualized on a SP8 confocal microscope (Leica) at 63X magnification. Images were quantified using Imaris cell imaging software version 9.9.1 (Bitplane, Oxford Instruments). For each image, a threshold for the red channel was established by selecting the lower intensity from the region of interest (plasma membrane, endosomes, or βarr2). The percentage of colocalization was determined by the percentage of the material from the red channel above threshold colocalized with the material from the green channel. To quantify the percentage of colocalization for confocal microscopy images, the “surfaces” module was selected isolating cells containing the region of interest for each image. Thresholds for each channel were established by selecting the lower intensity from the region of interest (plasma membrane, endosomes, or βarr2). Data are reported as red volume (red voxels) above the threshold that is co-localized with green volume (green voxels) above the threshold and reported as percentage.

### ELISA

To measure the relative cell surface expression of V_2_R and V_2_β_2_ (both tagged with a HA epitope at their amino-terminal), the same cell suspension containing DNA that was used for EbBRET assays was seeded in white 96-well plates (Greiner) previously coated with Poly-D-lysine at 30,000 cells/well (100 μl per well). Non transfected cells were used to establish the background of the assay. For the coating, Poly-D-Lysine solution (0.1 mg per ml; Cultrex) was added (50 μl per well) and the plates incubated at 37 °C for at least 30 minutes. Following the incubation, the solution was aspirated and wells washed two times with DPBS before adding the cell suspension containing DNA. 48 hours after seeding, cells were washed with DPBS and fixed by adding 50 μl per well of 4% paraformaldehyde in PBS (Thermo Scientific) and incubated at room temperature for 10 minutes. The fixing solution was aspirated and wells washed 3 times with the washing buffer (0.5% BSA in DPBS). The washing buffer was left in the wells for 10 minutes following the last wash. After the 10 minute incubation, the buffer was removed and 50 μl per well of monoclonal 3F10 anti-HA-Peroxidase (Sigma) 12.5 ng/ml in washing buffer was added and the plate incubated 1 hour at room temperature. The antibody was aspirated and wells washed 3 times with the washing buffer. The washing buffer was left in the wells for 10 minutes following the last wash and wells were washed again 3 times with DBPS only. After aspiration of the DPBS, 100 μl per well of SigmaFast^™^ OPD (Sigma) solution prepared as recommended by the manufacturer was added. Wells were incubated in presence of the OPD solution until the wells containing cells expressing receptors become yellow (typically 10 minutes). The reaction was stopped by addition of 25 μl per well of hydrochloride 3M in water. 100 μl per well were transferred to a transparent clear 96-well flat bottom plate (Corning) and absorbance at 492 nm was measured using a FLUOstar Omega microplate reader (BMG Labtech). The net absorbance represents the absorbance measured in presence of receptor minus the background (i.e. absorbance measured in absence of receptor).

### Data processing and statistical analyses

The data and statistical analyses comply with the recommendations on experimental design and analysis in pharmacology. In all experiments at least three independent experiments were performed and for each experiment. *n* value is provided in the corresponding figure legend. All experiments are performed in quadruplicates. A *P* value ≤0.05 was considered as statistically significant for all analyses. Normally distributed and normalized data to control for unwanted sources of variation are shown as mean ± standard error on mean (s.e.m). All statistical analyses and nonlinear regressions were performed using GraphPad Prism 9.4.1 software.

## Supplementary Material

1
