# Supplementary material for: β-arrestin-dependent and -independent endosomal G protein activation by the vasopressin type 2 receptor
Source: bioRxiv. 2023 Aug 21:2023.04.01.535208. Originally published 2023 Apr 2. Preprint. [Version 2] doi: 10.1101/2023.04.01.535208 (PMC10081317; doi:10.1101/2023.04.01.535208)
Supplement: Supplement 1 [file media-1.pdf]

Figure 1-figure supplement 1

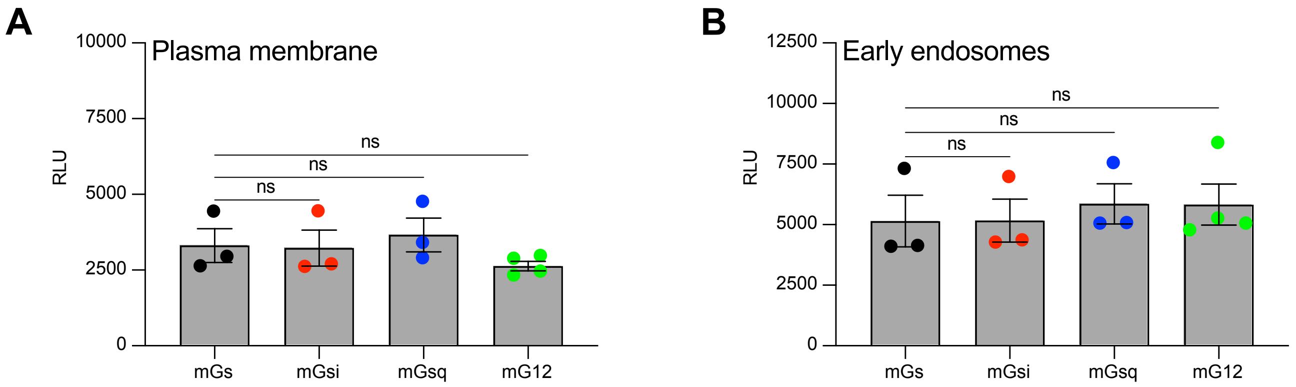

Equivalent expression of mG constructs for the V_2R kinetics

(A) Relative luminescence emitted by each of the four Rluc-fused mG constructs expressed by the cells used to monitor the kinetics of the recruitment of mG proteins at the plasma membrane upon V_2R stimulation. **(B)** Relative luminescence emitted by each of the four Rluc-fused mG constructs expressed by the cells used to monitor the kinetics of the recruitment of mG proteins at the early endosomes upon V_2R stimulation. n = 3 independent experiments for mGs, mGsi, and mGsq, and n = 4 for mG12. No statistical difference (ns) was detected between the expression of mGs and the other mG proteins using one-way ANOVA and Dunnett's post hoc test for multiple comparisons.
