# Supplementary material for: β-arrestin-dependent and -independent endosomal G protein activation by the vasopressin type 2 receptor
Source: bioRxiv. 2023 Aug 21:2023.04.01.535208. Originally published 2023 Apr 2. Preprint. [Version 2] doi: 10.1101/2023.04.01.535208 (PMC10081317; doi:10.1101/2023.04.01.535208)
Supplement: Supplement 3 [file media-3.pdf]

Figure 3-figure supplement 2

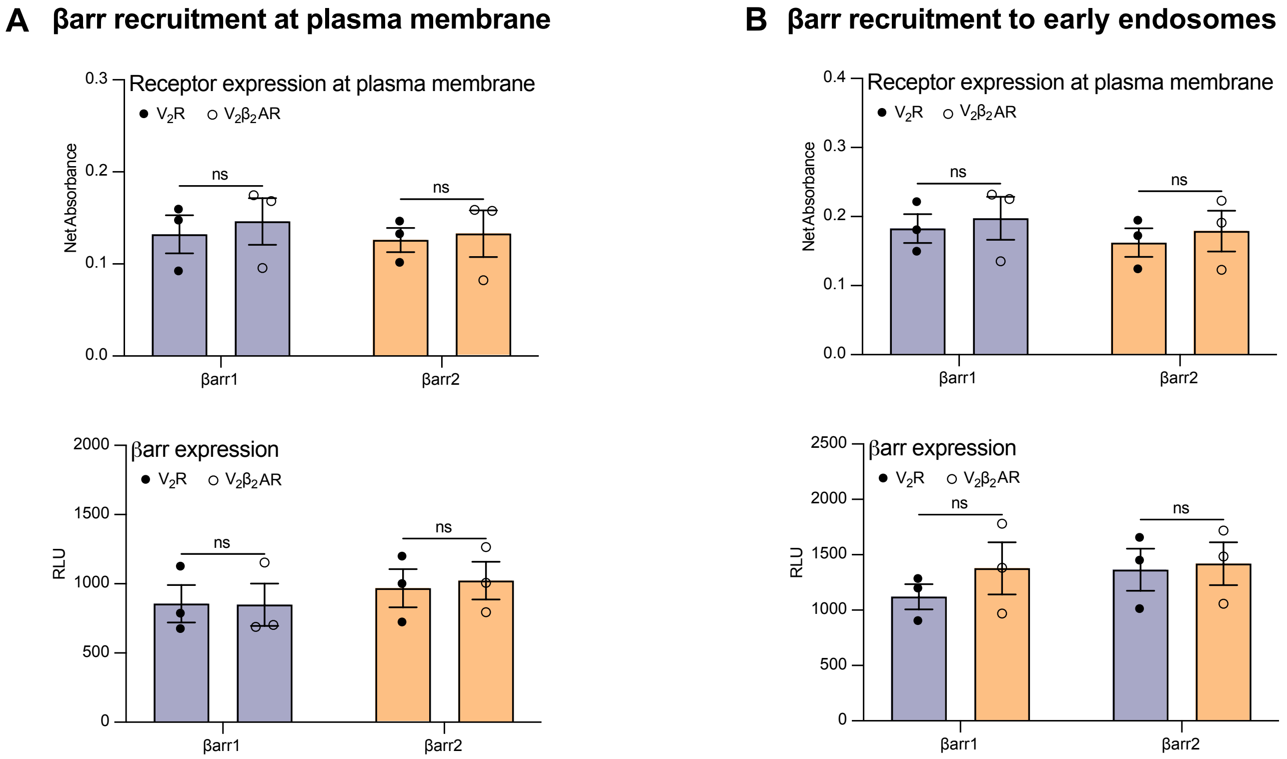

Relative expression of V_2R and $V_2\beta_2AR$ at the plasma membrane and of βarrs
(A) Relative V_2R and $V_2\beta_2AR$ expression at the plasma membrane (upper panel) and of βarr1 and βarr2 (bottom panel) in the βarr recruitment at the plasma membrane experiments. **(B)** Relative V_2R and $V_2\beta_2AR$ expression at the plasma membrane (upper panel) and of βarr1 and βarr2 (bottom panel) in the βarr recruitment at the early endosomes experiments. n = 3 biological replicates for all conditions. No statistical difference (ns) was detected between V_2R and $V_2\beta_2AR$ using two-way ANOVA and Sidak's post hoc test for multiple comparisons.
