# Supplementary material for: β-arrestin-dependent and -independent endosomal G protein activation by the vasopressin type 2 receptor
Source: bioRxiv. 2023 Aug 21:2023.04.01.535208. Originally published 2023 Apr 2. Preprint. [Version 2] doi: 10.1101/2023.04.01.535208 (PMC10081317; doi:10.1101/2023.04.01.535208)
Supplement: Supplement 4 [file media-4.pdf]

Figure 4-figure supplement 1

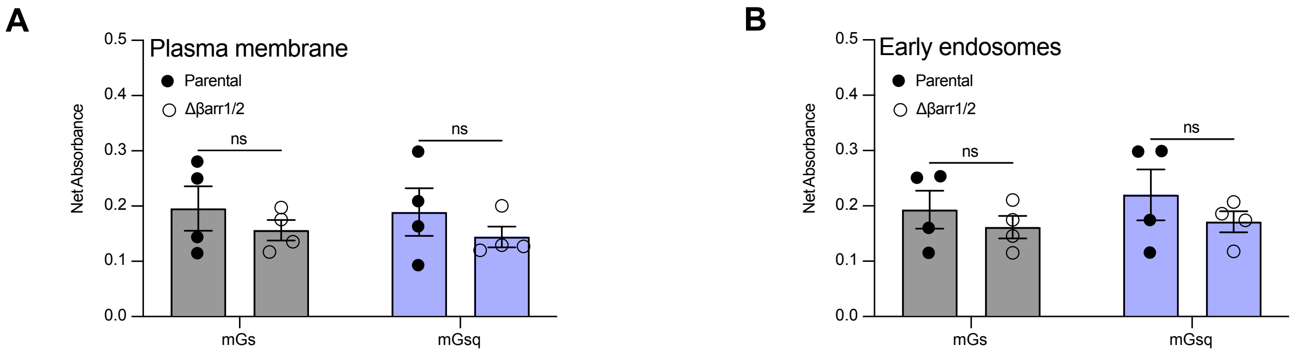

Relative expression of V_2R at the plasma membrane in parental and Δβarr1/2 cells

(A) Relative expression of V_2R in parental versus Δβarr1/2 cells determined by ELISA in the AVP dose-response curves of mGs and mGsq recruitment to the plasma membrane. **(B)** Relative expression of V_2R in parental versus Δβarr1/2 cells determined by ELISA in the AVP dose-response curves of mGs and mGsq recruitment to the early endosomes. n = 4 biological replicates for all experiments. No statistical differences (ns) were detected between parental and Δβarr1/2 cells as assessed by two-way ANOVA and Sidak's post hoc test for multiple comparisons.
