# Supplementary material for: β-arrestin-dependent and -independent endosomal G protein activation by the vasopressin type 2 receptor
Source: bioRxiv. 2023 Aug 21:2023.04.01.535208. Originally published 2023 Apr 2. Preprint. [Version 2] doi: 10.1101/2023.04.01.535208 (PMC10081317; doi:10.1101/2023.04.01.535208)
Supplement: Supplement 5 [file media-5.pdf]

Figure 4-figure supplement 2

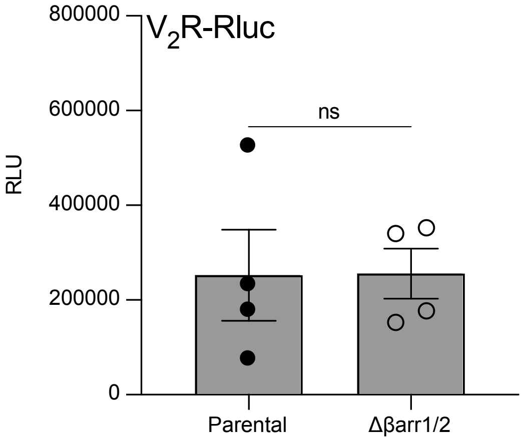

Relative expression of V_2R-Rluc in parental and Δβarr1/2 cells

Relative expression of V_2R-Rluc in parental versus Δβarr1/2 cells determined by monitoring the relative luminescence units (RLU) emitted by Rluc in the kinetics of V_2R internalization. *n* = 4 biological replicates. No statistical differences (ns) were detected between parental and Δβarr1/2 cells as assessed by a paired t test.
