# Supplementary material for: β-arrestin-dependent and -independent endosomal G protein activation by the vasopressin type 2 receptor
Source: bioRxiv. 2023 Aug 21:2023.04.01.535208. Originally published 2023 Apr 2. Preprint. [Version 2] doi: 10.1101/2023.04.01.535208 (PMC10081317; doi:10.1101/2023.04.01.535208)
Supplement: Supplement 7 [file media-7.pdf]

Figure 4-figure supplement 4

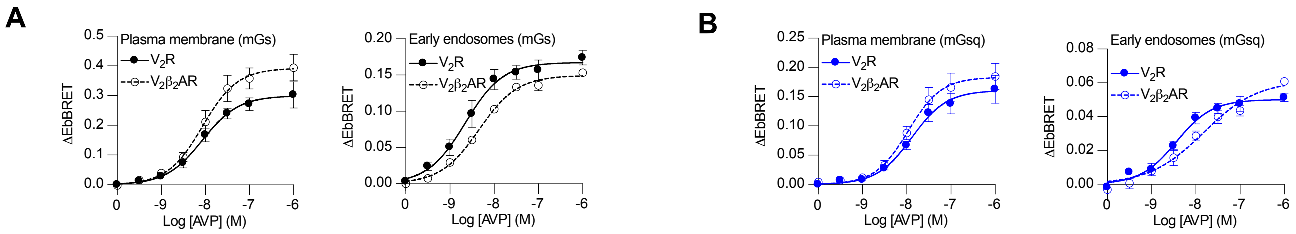

AVP dose-response curves of the recruitment of mGs and mGsq to the plasma membrane and early endosomes by the V_2R and $V_2\beta_2AR$

(A) Dose-dependent recruitment of mGs at plasma membrane (left panel) or early endosomes (right panel) in cells expressing V_2R or $V_2\beta_2AR$ upon 10 minutes (plasma membrane) or 45 minutes (early endosomes) of AVP treatment. **(B)** Dose-dependent recruitment of mGsq at plasma membrane (left panel) or early endosomes (right panel) in cells expressing V_2R or $V_2\beta_2AR$ upon 10 minutes (plasma membrane) or 45 minutes (early endosomes) of AVP treatment. *n* = 4 biological replicates for each condition.
