# Supplementary material for: β-arrestin-dependent and -independent endosomal G protein activation by the vasopressin type 2 receptor
Source: bioRxiv. 2023 Apr 2:2023.04.01.535208. Preprint. [Version 1] doi: 10.1101/2023.04.01.535208 (PMC10081317; doi:10.1101/2023.04.01.535208)
Supplement: Supplementary file 1 [file NIHPP2023.04.01.535208v1-supplement-1.pdf]

bioRxiv preprint doi: https://doi.org/10.1101/2023.04.01.535208; this version posted April 2, 2023. The copyright holder for this preprint (which was not certified by peer review) is the author/funder, who has granted bioRxiv a license to display the preprint in perpetuity. It is made available under aCC-BY 4.0 International license.

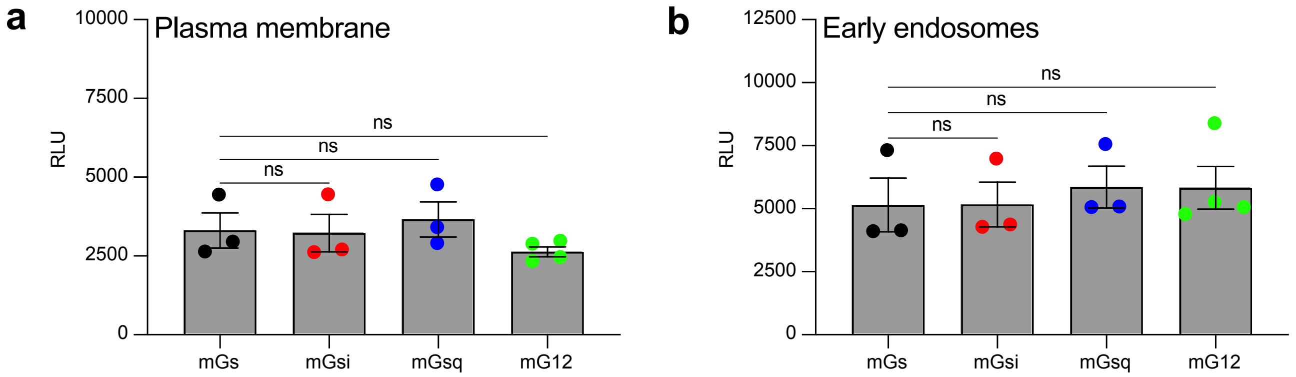

Supplementary Fig. 1 Scatter plots showing equivalent expression of Rluc-mG constructs for the kinetics experiments represented in Fig. 1a (a) and Fig. 1b (b). *n*=3 biological replicates for mGs, mGsi, and mGsq, and *n*=4 for mG12. Statistical differences between mGs and the other mG proteins were assessed by one-way ANOVA and Dunnett's post hoc test for multiple comparisons. No statistical differences were detected as compared to mGs (ns). In a, *P*=0.9985, 0.9130, 0.5902 for mGsi, mGsq, and mG12 respectively. In b, *P*=0.9999, 0.9072, 0.9019 for mGsi, mGsq, and mG12 respectively. The mean ± s.e.m are represented.

bioRxiv preprint doi: https://doi.org/10.1101/2023.04.01.535208; this version posted April 2, 2023. The copyright holder for this preprint (which was not certified by peer review) is the author/funder, who has granted bioRxiv a license to display the preprint in perpetuity. It is made available under aCC-BY 4.0 International license.

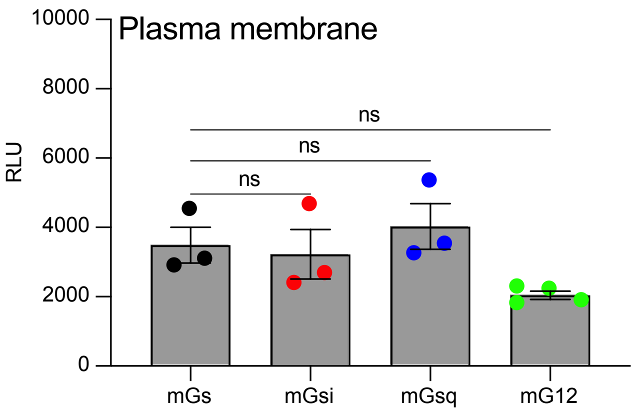

Supplementary Fig. 2 Scatter plots showing the relative expression of each Rluc-mG construct for the kinetics experiments represented in Fig. 2a. *n*=3 biological replicates for mGs, mGsi, and mGsq, and *n*=4 for mG12. Statistical differences between mGs and the other mG proteins were assessed by one-way ANOVA and Dunnett's post hoc test for multiple comparisons. No statistical differences were detected as compared to mGs (ns). *P*=0.9686, 0.8091, 0.1528 for mGsi, mGsq, and mG12 respectively. The mean ± s.e.m are represented.

bioRxiv preprint doi: https://doi.org/10.1101/2023.04.01.535208; this version posted April 2, 2023. The copyright holder for this preprint (which was not certified by peer review) is the author/funder, who has granted bioRxiv a license to display the preprint in perpetuity. It is made available under aCC-BY 4.0 International license.

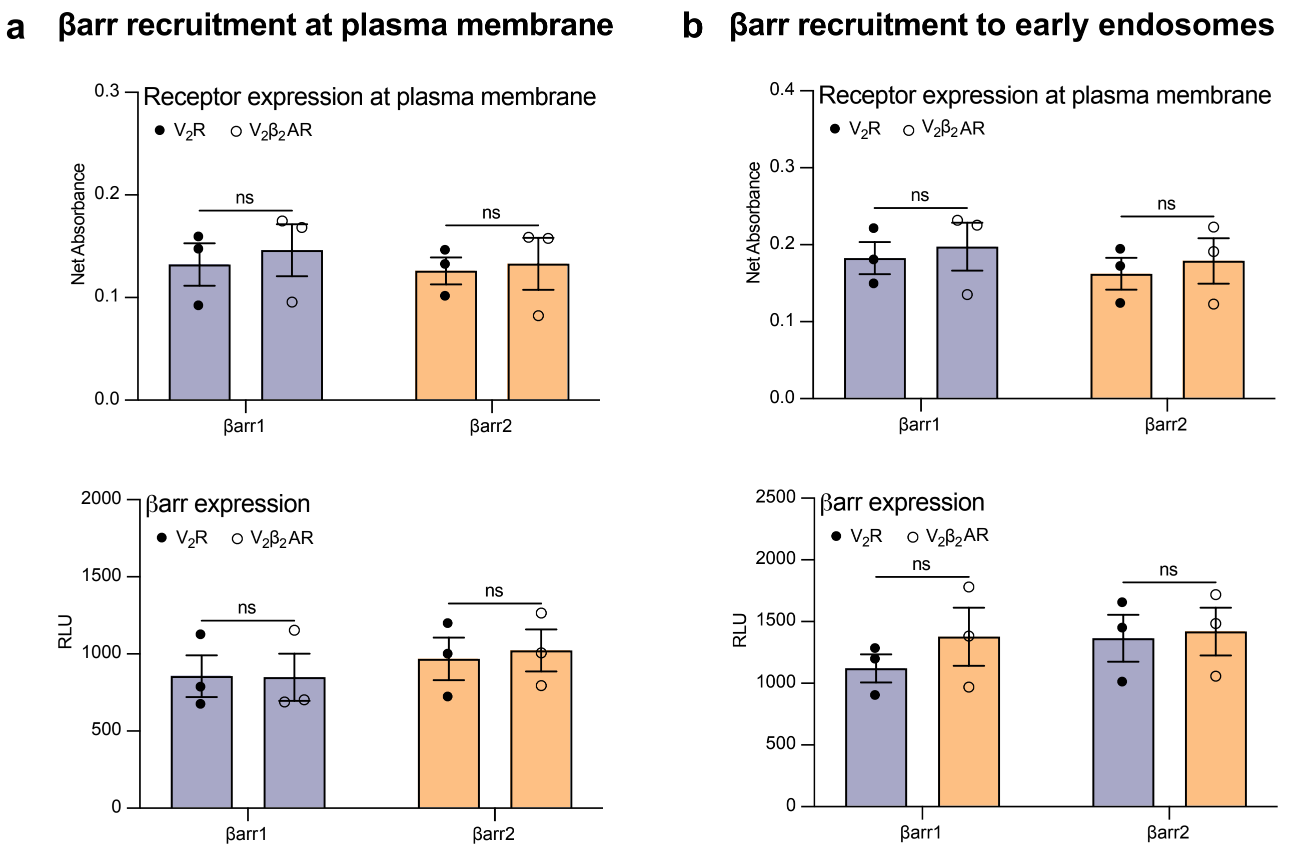

Supplementary Fig. 3 Relative expression of V_2R and $V_2\beta_2AR$ at the plasma membrane and of βarrs for the experiments represented in Fig. 2b. a,b, Scatter plots of relative V_2R and $V_2\beta_2AR$ expression at the plasma membrane (upper panels) and of βarr1 and βarr2 (bottom panels). n=3 biological replicates for each condition. Statistical differences between V_2R- and $V_2\beta_2AR$-expressing cells were assessed by two-way ANOVA and Sidak's post hoc test for multiple comparisons. No statistical differences were detected (ns). In a, upper panel, P=0.4485 for βarr1, and P=0.7995 for βarr2. In a, bottom panel, P=0.9730 for βarr1, and P=0.3188 for βarr2. In b, upper panel, P=0.6893 for βarr1, and P=0.6316 for βarr2. In b, bottom panel, P=0.0954 for βarr1, and P=0.8251 for βarr2. The mean ± s.e.m are represented.

bioRxiv preprint doi: https://doi.org/10.1101/2023.04.01.535208; this version posted April 2, 2023. The copyright holder for this preprint (which was not certified by peer review) is the author/funder, who has granted bioRxiv a license to display the preprint in perpetuity. It is made available under aCC-BY 4.0 International license.

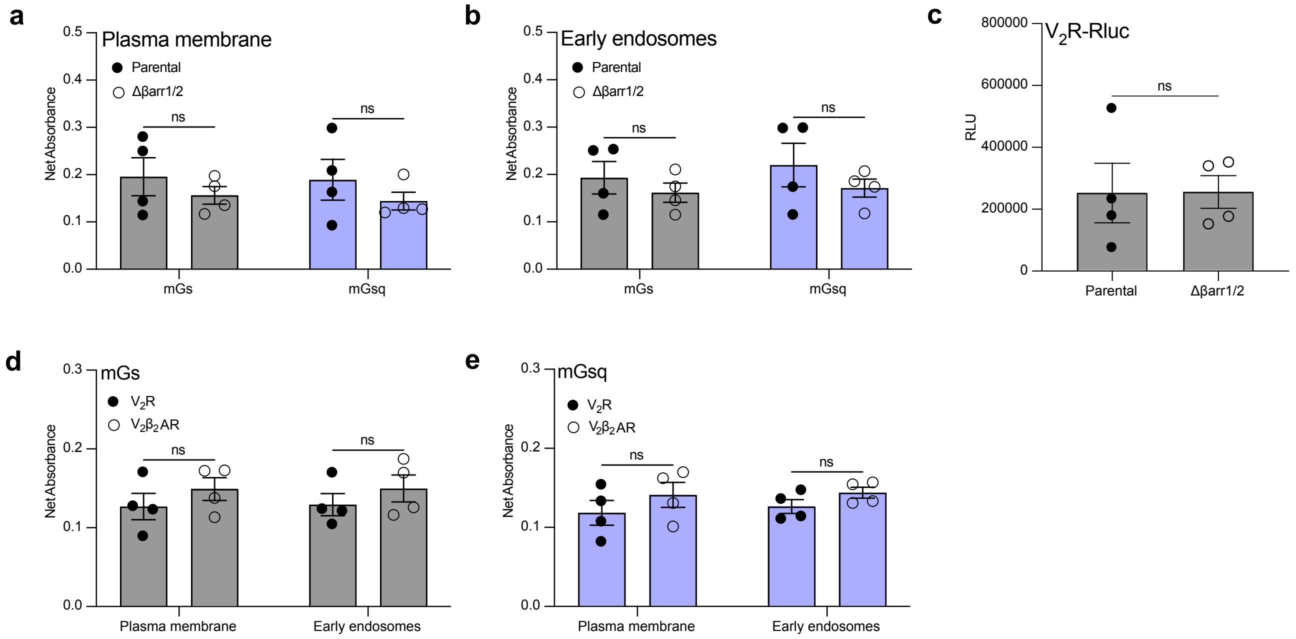

Supplementary Fig. 4 Relative receptor expression related to the experiments in Fig. 3. a,b Scatter plots of relative expression of V_2R in parental versus Δβarr1/2 cells used to measure mGs and mGsq recruitment to the plasma membrane and early endosomes in Fig. 3a,b. Statistical differences between parental and Δβarr1/2 cells were assessed by two-way ANOVA and Sidak's post hoc test for multiple comparisons. No statistical differences were detected (ns). In a, *P*=0.5785 for mGs, and *P*=0.4970 for mGsq. In b, *P*=0.7238 for mGs and *P*=0.4882 for mGsq. **c,** Scatter plots showing the relative expression of V_2R-Rluc in parental versus Δβarr1/2 cells used to monitor V_2R internalization in Fig. 3c. Relative V_2R-Rluc expression was assessed by monitoring the relative luminescence units (RLU) emitted by the luciferase. No statistical differences were detected (ns) using a paired t test (*P*=0.9748). **d,e,** Scatter plots showing the relative expression of V_2R and $V_2\beta_2$AR at the plasma membrane in the cells used to determine the transduction coefficients represented in Fig. 3d,e. Statistical differences between the V_2R and $V_2\beta_2$AR were assessed by two-way ANOVA and Sidak's post hoc test for multiple comparisons. No statistical differences were detected (ns). In d, *P*=0.0996 for the plasma membrane, and *P*=0.1242 for early endosomes. In e, *P*=0.1615 for the plasma membrane, and *P*=0.2944 for early endosomes. *n*=4 biological replicates for all conditions. The mean ± s.e.m are represented.

bioRxiv preprint doi: https://doi.org/10.1101/2023.04.01.535208; this version posted April 2, 2023. The copyright holder for this preprint (which was not certified by peer review) is the author/funder, who has granted bioRxiv a license to display the preprint in perpetuity. It is made available under aCC-BY 4.0 International license.

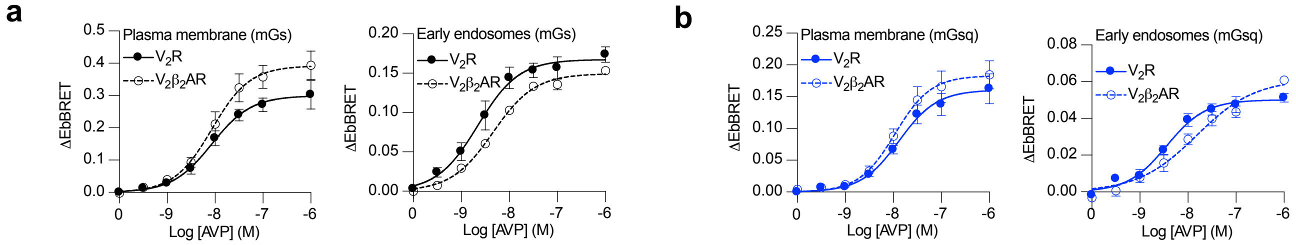

Supplementary Fig. 5 AVP dose-response curves for the recruitment of mGs and mGsq to the plasma membrane and early endosomes by the V$_2$R and V$_2$β$_2$AR. a,b, Dose-dependent recruitment of mGs and mGsq in HEK293 cells upon 10 minutes (plasma membrane) or 45 minutes (early endosomes) of AVP treatment. See Supplementary Table 2 for dose-response parameters and Supplementary Fig. 4d,e for relative expression of the V$_2$R and V$_2$β$_2$AR at the plasma membrane. *n*=4 biological replicates for each condition and the mean ± s.e.m are represented.

bioRxiv preprint doi: https://doi.org/10.1101/2023.04.01.535208; this version posted April 2, 2023. The copyright holder for this preprint (which was not certified by peer review) is the author/funder, who has granted bioRxiv a license to display the preprint in perpetuity. It is made available under aCC-BY 4.0 International license.

			AVP-induced maximal efficacy ΔEbBRET ± SEM	Potency $LogEC_{50}$ ± SEM
Plasma membrane	mGs	Parental	0.324 ± 0.006	-7.90 ± 0.03
		Δβarr1/2	0.376 ± 0.011**	-7.99 ± 0.04
	mGsq	Parental	0.168 ± 0.005	-7.81 ± 0.04
		Δβarr1/2	0.180 ± 0.009	-7.95 ± 0.07
Early endosomes	mGs	Parental	0.180 ± 0.004	-8.37 ± 0.03
		Δβarr1/2	0.074 ± 0.004****	-8.34 ± 0.09
	mGsq	Parental	0.050 ± 0.002	-8.38 ± 0.08
		Δβarr1/2	0.024 ± 0.002****	-8.32 ± 0.12

Supplementary Table 1 Parameters related to AVP dose-response curves of the mGs and mGsq recruitment to the plasma membrane or early endosomes in parental and Δβarr1/2 cells. ΔEbBRET values from Fig. 3a,b were fitted using four parameters equation with the bottom fixed at zero. *n*=4 biological replicates for each condition. Statistical differences for AVP-induced maximal efficacy (ΔEbBRET) and potency ($LogEC_{50}$) between parental and Δβarr1/2 cells were assessed by comparing independent fits with a global fit that shares the selected parameter using extra sum-of-squares F test (**≤ 0.01, ****≤ 0.0001). The mean ± s.e.m are represented.

bioRxiv preprint doi: https://doi.org/10.1101/2023.04.01.535208; this version posted April 2, 2023. The copyright holder for this preprint (which was not certified by peer review) is the author/funder, who has granted bioRxiv a license to display the preprint in perpetuity. It is made available under aCC-BY 4.0 International license.

			AVP-mediated maximal efficacy ΔEbBRET ± SEM	Potency $LogEC_{50}$ ± SEM	Transduction coefficient Log(τ/Ka) ± SEM
mGs	Plasma membrane	V_2R	0.300 ± 0.018	-8.07 ± 0.10	7.98 ± 0.05
		$V_2\beta_2AR$	0.392 ± 0.025***	-8.07 ± 0.10	8.04 ± 0.08
	Early endosomes	V_2R	0.167 ± 0.008	-8.65 ± 0.09	8.63 ± 0.11
		$V_2\beta_2AR$	0.150 ± 0.003	-8.34 ± 0.04**	8.31 ± 0.02*
mGsq	Plasma membrane	V_2R	0.161 ± 0.011	-7.88 ± 0.11	7.90 ± 0.10
		$V_2\beta_2AR$	0.183 ± 0.013	-7.97 ± 0.11	7.99 ± 0.10
	Early endosomes	V_2R	0.050 ± 0.002	-8.44 ± 0.08	8.31 ± 0.03
		$V_2\beta_2AR$	0.061 ± 0.005*	-7.82 ± 0.14***	7.91 ± 0.17*

Supplementary Table 2 Parameters related to AVP dose-response curves of the mGs and mGsq recruitment to the plasma membrane or early endosomes in cells expressing the V_2R or $V_2\beta_2AR$. ΔEbBRET values from Supplementary Fig. 5 were fitted using four parameters equation with the bottom fixed at zero. *n*=4 biological replicates for each condition. Statistical differences between AVP-induced maximal efficacy and potency of mGs or mGsq recruitment by V_2R and $V_2\beta_2AR$ were assessed by comparing independent fits with a global fit that shares the selected parameter using extra sum-of-squares F test (**≤0.01, ***≤ 0.001). Statistical significance for Log(τ/Ka) values from V_2R-expressing cells compared to cells expressing the $V_2\beta_2AR$ were assessed by two-way ANOVA and Sidak's post hoc test for multiple comparisons (*≤0.05). The mean ± s.e.m are represented.
